# Unchain My Heart: Integrins at the Basis of iPSC Cardiomyocyte Differentiation

**DOI:** 10.1155/2019/8203950

**Published:** 2019-02-13

**Authors:** Rosaria Santoro, Gianluca Lorenzo Perrucci, Aoife Gowran, Giulio Pompilio

**Affiliations:** ^1^Unità di Biologia Vascolare e Medicina Rigenerativa, Centro Cardiologico Monzino IRCCS, via Carlo Parea 4, Milan, Italy; ^2^Dipartimento di Scienze Cliniche e di Comunità, Università degli Studi di Milano, via Festa del Perdono 7, Milan, Italy

## Abstract

The cellular response to the extracellular matrix (ECM) microenvironment mediated by integrin adhesion is of fundamental importance, in both developmental and pathological processes. In particular, mechanotransduction is of growing importance in groundbreaking cellular models such as induced pluripotent stem cells (iPSC), since this process may strongly influence cell fate and, thus, augment the precision of differentiation into specific cell types, e.g., cardiomyocytes. The decryption of the cellular machinery starting from ECM sensing to iPSC differentiation calls for new *in vitro* methods. Conveniently, engineered biomaterials activating controlled integrin-mediated responses through chemical, physical, and geometrical designs are key to resolving this issue and could foster clinical translation of optimized iPSC-based technology. This review introduces the main integrin-dependent mechanisms and signalling pathways involved in mechanotransduction. Special consideration is given to the integrin-iPSC linkage signalling chain in the cardiovascular field, focusing on biomaterial-based *in vitro* models to evaluate the relevance of this process in iPSC differentiation into cardiomyocytes.

## 1. Introduction

The integrin protein family is a large group of transmembrane receptors, particularly involved in cell-extracellular matrix (ECM) proteins and cell-cell adhesion. Moreover, integrins constitute an important and functional bridge between the ECM and the cytoskeleton and are able to activate several intracellular signalling pathways. After the first report of their identification [1, 2], in the last 30 years, how the integrin protein family assumed a key role in mechanotransduction biology, particularly as mediators of a bidirectional signalling mode, has been extensively reported. Integrins are able to read and transmit signals from the extracellular microenvironment to the internal cellular *milieu*, including the cytoplasm and nucleus (outside-in), leading to a cellular reaction that may alter cell behaviour and/or also the composition of the ECM (inside-out). Several downstream mechanisms of integrins activate biochemical signalling cascades which have impact on different cell functions by regulating crucial molecular pathways involved in cell survival, proliferation, motility, and differentiation, both in physiological and pathological scenarios [3–6].

In 2007, the groundbreaking discovery of a universal protocol to reprogram mammalian somatic cells into induced pluripotent stem cells (iPSC) [7], made by Takahashi and colleagues, brought immense potential to the fields of regenerative and personalized medicine. In fact, these cells can differentiate into cell types from all the three developmental germ layers: ectoderm, mesoderm, and endoderm. iPSC-derived cells have modelled, previously unreproducible, human diseases, e.g., long QT (LQT) syndrome [8], and have already been used in two clinical trials for age-related macular degeneration [9] and advanced heart failure [10].

The efficacy of iPSC as a model system for the study of the molecular mechanisms guiding pathological development is tightly linked to the success of *in vitro* simulation of the environmental cues responsible for cell fate *in vivo*. Mechanosensing-mediated pathways are relevant not only for enhancing iPSC reprogramming efficiency [11] but also for supporting iPSC-derived cardiomyocyte (iPSC-d-CM) maturation [12]. Thus, several techniques have been proposed for the design of substrates regulating integrin activation by tuning chemical, geometrical, or mechanical parameters. The last part of this review is dedicated to the discussion of these methods and their relevance to spearheading the clinical translation of the iPSC technology.

## 2. Integrin Structure, Extracellular Ligands, and Focal Adhesion (FA) Complexes

The integrin family was firstly identified by using antibodies against integrin *β* subunits which unveiled several coimmunoprecipitating proteins. Integrin heterodimers are composed of noncovalently associated *α* and *β* subunits [3]. The heterodimeric structure and functionality of these receptors were made clear only after the use of specific peptides, e.g., arginine-glycine-aspartic acid (Arg-Gly-Asp; RGD tripeptide) and integrin *α* subunit-recognizing antibodies. To date, it is well known that the integrin family is constituted by 18 *α* subunits and 8 *β* subunits, possibly assembled in 24 different heterodimers [13]. Depending on integrin subunit composition, these molecules show specific extracellular ligand properties and can be classified into 4 main subgroups [14] listed in Table 1. This feature implies that the expression pattern of integrins is tissue specific [3]. In addition to a large extracellular domain, each heterodimer also has a transmembrane domain and a short cytoplasmic domain, which forms a fundamental functional link with the cytoskeleton [14].

As shown in Table 1, cells expressing *β*1 integrin are generally outbound to collagen when associated with *α*1, *α*2, *α*10, and *α*11 subunits. Otherwise, when *β*1 integrin is bound to laminin, it is complexed with *α*3, *α*6, and *α*7 subunits. Depending on which specific *α* subunit it heterodimerizes with, *β*1 integrin recognizes the RGD motif (*ανβ*1, *α*5*β*1, or *α*8*β*1) or leukocyte-specific receptors (*α*9*β*1, *α*4*β*1). All integrin heterodimers containing *α*ν subunits are specifically associated with substrates with the RGD sequence. Similarly, all integrins expressing *β*2 subunit are members of the leukocyte-specific receptor-binding integrin subgroup. Lastly, *α*6*β*4 integrin belongs to the previously mentioned laminin receptor binding group [15].

It has been reported that *β*1 subunit-containing integrins, such as *α*5*β*1, are predominantly recruited to the leading edge of cells moving on a 2D surface [16], whereas *β*3 subunit-containing integrins are responsible for the increase in the number of focal adhesions (FA) and cell spreading area because of its role in structural reinforcement of adhesion [17, 18].

**Figure 1:**
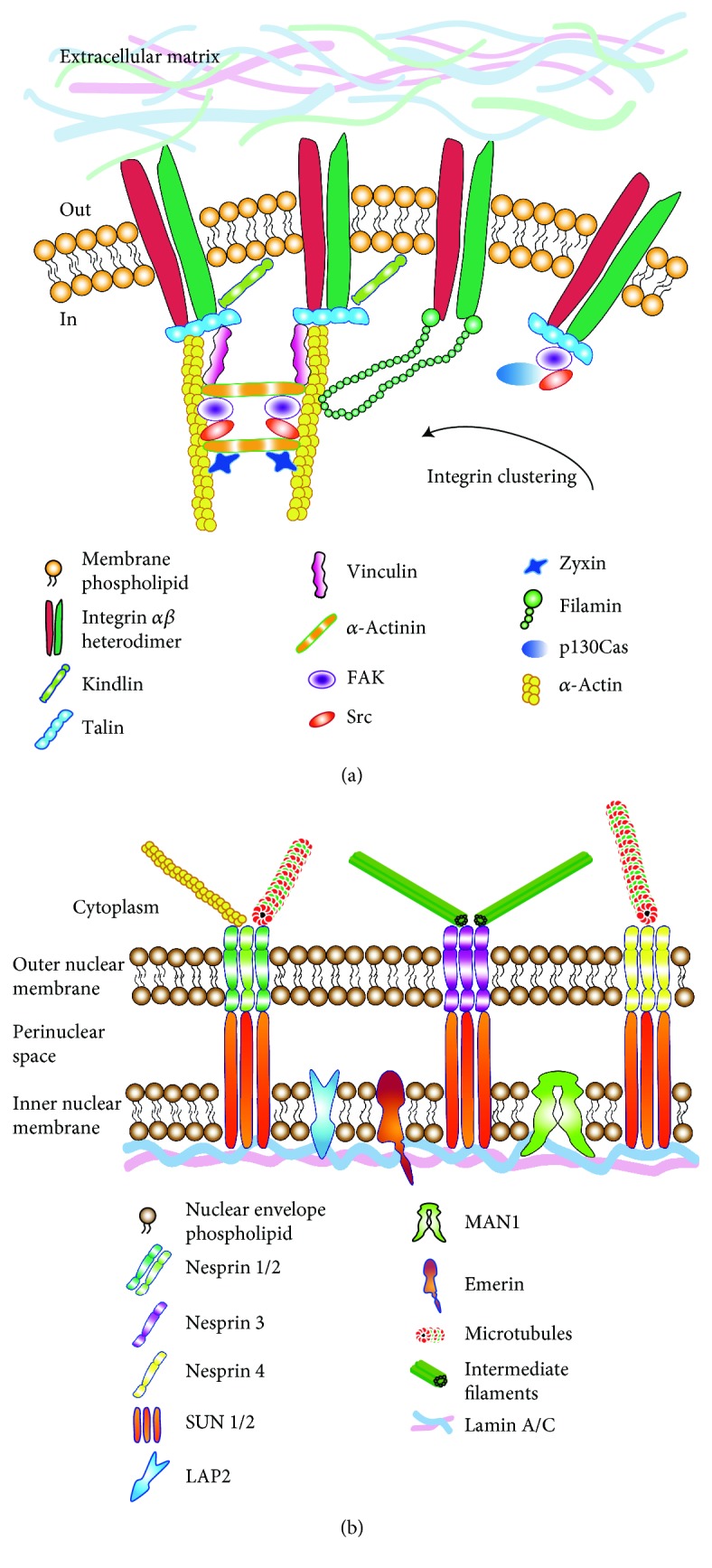
Cytoplasmatic membrane and nuclear envelope mechanotransduction protein complexes. (a) The image depicts the main mediators involved in the mechanotransduction chain, starting from the integrin subunits, specifically binding ECM compounds, to cytoskeleton polymerization, through the activity of focal adhesion effectors. (b) The figure summarizes the link between cytoskeleton and nuclear lamin A/C, through the nuclear envelope complexes, responsible for the gene expression modulation downstream to mechanotransduction.

Since integrins work as receptors of several ECM components, they strongly contribute individually to FA-mediated signalling and rigidity sensing by mechanically changing their structural conformation. In external force mechanosensing, this integrin function can be considered as a primary step, followed by a series of secondary mechanosensor activities, which respond intracellularly to force-dependent alterations. Thus, extracellular tension transmitted through integrins elicits the binding of several intracellular elements, which in turn activate themselves and strengthen the integrin connection to actin (Figure 1(a)). Among these intracellular factors, the main ones involved in mechanotransduction are talin, vinculin, kindlin, *α*-actinin, zyxin, filamin, and p130Cas. The interaction of integrin cytoplasmic tails with one of these adaptor proteins, such as talin, is the main mechanism leading to full integrin activation. For example, talin, together with vinculin, plays a crucial role in the force-dependent stabilization of FA by changing its conformation after tension. Talin binds through its FERM (four-point-one protein/ezrin/radixin/moesin) domain to the NPxY amino acid motifs on integrin tails, inducing their activation [19–21]. Vinculin presents a self-inhibited state, exerted by its head and tail domain interaction [22], and becomes activated after its tail domain binds to *α*-actinin alone or together with actin and phosphatidyl-inositol-4,5-bisphosphate (PIP2) [23–26]. An increase in cellular tension, which is strictly related to ECM stiffness and cytoskeletal recruitment, stabilizes vinculin in the activated conformation, and leads to its FA recruitment [27, 28]. Another cooperating adaptor protein is kindlin which contributes to integrin activation [29, 30]. Until this link of the mechanotransduction chain, talin and vinculin act as direct mechanical sensors, able to feel ECM properties, while *α*-actinin, a spectrin superfamily component important for the structural organization of the cell, also provides a scaffold to connect the mechanotransduction chain with the previously mentioned downstream effectors [31]. Thus, *α*-actinin is an indirect link in the mechanotransduction chain. For this reason, cell stretching could result in the dissociation of several proteins that are weakly bound to *α*-actinin at multiple sites [32]. Among these weakly bound proteins, zyxin binds a central region of *α*-actinin [33]; when a certain type of mechanical stimulus occurs, this molecule translocates from FA to stress fibres [34]. Other connecting proteins between integrins and actin such as filamins [35] play a mechanoprotective role by stabilizing the actin cytoskeleton through linkage to the cytoplasmatic membrane. In fact, under mechanical stress, filamin domains change their conformational status considerably [36]. This event leads to extension and reversible unfolding [37], which allows filamin stretching and subsequent protection of the linkage between F-actin and the cytoplasmatic membrane [38]. Interestingly, force applied through clustered *β*1 integrins leads to the transcriptional upregulation of filamin A [39].

Lastly, Src family kinase p130Cas (Cas: Crk-associated substrate) contains a N-terminal SH3 domain, which binds the polyproline motifs of the tyrosine kinases, FA kinase (FAK) [40, 41], and other proteins such as vinculin [42]. The SH3 domain is followed by a large substrate domain with 15 repetitions of the YxxP motif (where x is any amino acid), which is a main site of tyrosine phosphorylation on the Cas molecule [43]. Once phosphorylated, the p130Cas SH3 domain serves as a docking site for the SH2 domains of Crk or Nck adaptor proteins [44, 45]. In nonadherent cells, p130Cas is localized in the cytoplasm and, after integrin receptor activation, translocates to FA where the phosphorylation of substrate domain tyrosine residues takes place [46]. p130Cas activation after integrin engagement regulates the reorganization of the actin cytoskeleton and cell processes, such as spreading and migration [47]. Moreover, this tyrosine phosphorylation triggers signalling pathways leading to the regulation of cell survival and proliferation [48, 49]. Recently, investigators highlighted how p130Cas is able to influence actin remodelling and concomitant muscle-specific gene expression [42, 50].

### 2.1. Mechanical Stress-Reactive Nuclear Complexes

Based on the previous discussion, it can be said that cells perceive, adapt themselves to, and modify the ECM microenvironment physical features by using specific protein structures including the mechanosensing machinery of cell-ECM and cell-cell interactions, secondary mechanosensors, and different mechanotransduction pathways. Interestingly, this mechanism is mediated by a direct effect of mechanical linkage which is specific and sufficient to transmit the extracellular stimuli into the nuclei [42, 54, 55].

The strong and intimate relationship between integrins, FA, actin cytoskeleton, and nuclear structures has been well documented in the last years. Several lines of evidence report that actin fibres communicate the mechanical properties of the internal cellular environment to the nucleus and consequently strongly affecting gene regulation and expression [56, 57]. The nucleus contains a stratified network of mediators, linking the nuclear envelope to the nucleoskeleton and chromatin (Figure 1(b)). Structural alterations of nuclei are responsible for gene modulation of multiple mediators such as those related to mechanotransduction and differentiation [58]. The nuclear lamina consists in filamentous lamin proteins (lamins A, B, and C) that form the mechanical support of the inner nuclear membrane. Several other membrane proteins, including LAP2, emerin, and MAN1, are essential nuclear constituents [59]. To date, it is well known that the cytoskeleton is strongly linked with the nuclear lamina [60]; nevertheless, most of the current information is derived from studies on isolated nuclei [61]. Two distinct protein families, the SYNE/nesprin family and the SUN family [62] colocalize in the nuclear membrane and are connected both with cytoskeleton and nuclear lamina. Studies on *C. elegans* revealed that homologues of nesprin 2 and SUN1/2 were associated with actin, at their N- and C-terminals, respectively. For this reason, the term LINC was coined, indicating that these protein structures were linkers of nucleoskeleton and cytoskeleton [63, 64]. Every molecular component of this important complex shows distinct binding peculiarity; while nesprins 1 and 2 are specialized in actin, microtubule, and kinesin binding, on the other hand, nesprins 3 and 4 are able to bind intermediate filaments and microtubules, respectively [65–67]. Concerning the SUN protein family, the oligomerization as a trimer of these molecules is strongly required for nesprin binding [68]. These molecular events, which were experimentally observed on isolated nuclei, suggested their effectiveness in whole cell systems, thus supporting their contribution to mechanical cues. Thus, isolated nuclei react to the physical forces in a similar manner to complete cells, because of the presence of LINC complex, by which nuclei display adhesion ability acting as force-sensitive signalling hubs for cytoplasmic proteins and tuning nuclear responses to various mechanosensory inputs [61]. Finally, among LINC complex members, emerin plays a strategic role on the inner nuclear membrane, since it can be phosphorylated by Src kinases after a tension stimulus applied on isolated nuclei through nesprin 1 [61]. This event overlaps lamin A/C accumulation, which leads to the strengthening of the nuclear membrane. It is important to point out that Emery-Dreifuss muscular dystrophy is predominantly due to emerin gene mutations [69]; moreover, cells derived from emerin knockout transgenic mice show mechanotransduction impairments [62, 70].

### 2.2. Mechanosensing Signalling Pathways

The major chemical signals elicited by mechanical stress at the cell surface are as follows: (i) calcium influx through cation channels activated by stretch stimuli, (ii) activation of nuclear factor kappa-B (NF-*κ*B), (iii) stimulation of mitogen-activated protein kinases (MAPKs), and (iv) changes in the activity of small GTPases, e.g., Ras, Rac1, and RhoA [71–79].

Peculiar mechanisms have been unveiled, e.g., the adaptor protein p130Cas which is physically stretched in response to applied force both *in vivo* and *in vitro*. This stimulus exposes the previously masked phosphorylatable sites of p130Cas [80], that are substrates for Src family kinases which trigger further downstream responses [81].

Among the previously mentioned signalling pathways, there are two cascades which are strongly involved in the context of integrin-mediated mechanotransduction, namely, Rac1 and RhoA. In fact, these two members of the small Rho family GTPases regulate actin assembly and contraction [82, 83]. While active Rac1 controls actin polymerization at the leading edges of motile cells and is involved in lamellipodia focal complex formation, active RhoA is necessary for stress fibre formation. Notably, the two main RhoA downstream effectors are the diaphanous-related formin protein mDia1, a formin family member that serves as an actin nucleating factor and so facilitates actin polymerization and assembly [42, 84–86] and the protein kinases ROCK-1 and ROCK-2, which promote actin contraction mediated by nonmuscle myosin-II [82, 83].

**Figure 2:**
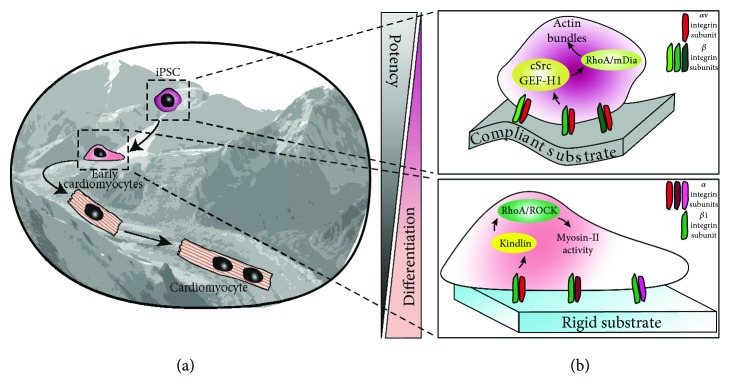
Integrin expression in iPSC at different stages of differentiation. The picture on (a), inspired by the Waddington diagram and John Piper's original artwork [103], represents iPSC undergoing cardiomyocyte differentiation. During these early stages, cells lose their potency, acquiring, in parallel, cardiomyocyte features. This process is linked to a specific integrin expression, further exacerbated by the growing substrates. As displayed in (b), cells with a higher potency and a lower degree of differentiation express, on compliant substrates, a higher amount of integrin heterodimers, preferentially containing *αν* integrin subunits. On the other hand, iPSC on rigid substrates lose potency in favour of differentiation and express integrins with *β*1 integrin subunits.

One important feature of Rac1 and RhoA is that each of these two mediators negatively regulates the other, leading to a discrimination of the activity in certain cell subareas [82], as depicted in Figure 2. Indeed, while some cell regions show higher Rac1 activity responsible for moving and protrusion, other subareas of the same cell with higher RhoA activity are more concentrated on adhesion and contraction. Several studies addressed the strong relationship between the integrin-dependent mechanical stress and the sustained modulation in Rac1 and RhoA activity [87–91]. As previously mentioned, several information on different features of small GTPase response to integrin-derived mechanical stimulation are, to date, still missing, e.g., the exact signalling time course and the types of cells and stresses involved. However, it is well established that mechanical stimulation leads to small GTPase activity driving the cell to undergo a strong and complex actin cytoskeleton remodelling which also influences the adhesion features [92].

The main molecular events leading to the activation of these numerous pathways are considerably distinct and depend on the signal being activated. Activation of tyrosine kinases and protein tyrosine phosphorylation play crucial roles in the assembly and turnover of FA, as well as in mechanotransduction. FAK and members of the Src family are key tyrosine kinases, controlling FA functions and complex stability [93]. After integrin engagement, FAK is recruited to adhesion areas to provide both scaffolding and kinase activity. The autophosphorylation of FAK defines a docking site for Src kinase, which subsequently phosphorylates FAK on multiple tyrosine residues. All these events are helpful for full FAK activation [94]. Following this, the FAK-Src complex recruits the p130Cas protein and several other adaptor proteins, phosphorylating multiple residues on their sequence. After FAK autophosphorylation and the generation of docking substrates for the SH2 domains of the adaptor protein Crk (also known as p38), this interaction leads to the activation of downstream signalling cascades, such as Rac1 GTPase, previously reported as the pathway involved in actin polymerization and the formation of new focal complexes at the leading edge of the cell [95, 96]. It has been demonstrated that inhibitors of actomyosin contractility lead to a loss of tension due to a rapid dismantling of FA [23, 27]. Mechanical tension activates guanine nucleotide exchange factors (GEFs) for Rho, such as Vav2, GEF-H1, or LARG, which subsequently induce GTP loading of Rho. This leads, in turn, to the activation of downstream effectors ROCK and mDia. Furthermore, several integrins containing *β*1 subunits (e.g., *α*5*β*1) activate the pathway mediated by Rho-ROCK-myosin-II to induce forces mediated by actomyosin (Figure 2), whereas *αν* subunit-containing integrins, e.g., *ανβ*3 and *ανβ*5, are more involved in external force adaptation and regulate both the stress fibre synthesis and FA area expansion through the pathway mediated by Rho-mDia [97]. ROCK-mediated activation of myosin light chain, together with inhibition of myosin light chain phosphatase, rapidly increases myosin-II activity and actomyosin contractility (Figure 2). Overall, ROCK activity leads to actin stress fibre stabilization.

## 3. Integrins and iPSC

Human embryonic stem cells (ESC) and iPSC, obtained by somatic cell reprogramming, are promising pluripotent stem cells, with the potential for recapitulating monogenic diseases and producing cell-based therapies [98]. In order to maximize the full potential of these cells, it is mandatory to enhance investigation and knowledge on the best culture conditions able to maintain plasticity, self-renewal, and external stimuli responsiveness as well as attenuate cell death events. Despite the lack of knowledge, there is increasing evidence regarding the contribution of integrins on pluripotent cell-ECM interaction [98–101].

The intricate and incompletely understood nature of cell fate and potency routes was succinctly represented in the self-acknowledged oversimplified [102] Waddington diagram [103] which is based on original artwork created by John Piper [104]. We have reimagined this iconic diagram to better summarize integrin engagement for cell-cell and cell-ECM binding and how these interactions affect cell fate (Figure 2). Mechanisms governing the transition from a somatic cell to an iPSC, which is initiated by the expression of exogenously acquired transcription factors, are continuously evolving, and much effort is directed to optimize the stochastic process of cell fate rewinding, in order to achieve a fully predictable process [105]. Reprogramming involves three sequential steps: initiation, stabilization, and maturation [106]. Indeed, molecular mediators, e.g., microRNA, or biophysical cues, e.g., nanotopography, can supplement or replace some of the classical reprogramming factors commonly used to enhance reprogramming efficiency [107–113].

In this context, the activation of the transforming growth factor-*β* (TGF-*β*) pathway and the expression of E-cadherin are of interest. While the former is a potent inhibitor of mesenchymal-to-epithelial transition, which is essential for a successful reprogramming [114], the latter is not only important for the maintenance of pluripotency and proper colony morphology but is also an absolute requirement for iPSC generation as well as the primary gatekeeper to the differentiation progression [115]. Moreover, iPSC kinome-wide functional analysis during reprogramming found a critical role in the cytoskeletal remodelling process. Specifically, the key serine/threonine kinases, testicular protein kinase-1 and LIM kinase-2, phosphorylate the actin-binding protein cofilin to modulate the cell reprogramming process.

Attempts have been made to transfer from the traditional 2D culture of iPSC to 3D culture in large-scale bioreactors, a step which would facilitate iPSC culture on industrially and clinically relevant scales [116, 117]. Since iPSC normally exist in tightly packed colonies, their dissociation into single cells, which is needed to ensure uniform cell distribution and diffusion of treatments, is a major stressor and initially caused high rates of cell death before the routine use of ROCK inhibitors during passaging [118]. Indeed, approaches that prevent actin-myosin contraction, such as downregulation of myosin heavy or light chains and ROCK inhibition [119], protect cells from cell death processes. Moreover, it has been demonstrated that a direct inhibition of Rho-ROCK-myosin-II activation involving E-cadherin leads to a uniform differentiation of pluripotent stem cell colonies [115].

To date, the generation of iPSC in two parallel states of pluripotency has been described: naïve and primed [120]. Naïve iPSC are considered closer to a ground state, similar to preimplantation epiblasts, while primed iPSC correspond to cells found in the postimplantation epiblasts which are ready or “primed” to differentiate [121–123]. The importance of the pluripotency state is crucial to understand and to harness in research field, as it is currently appreciated that naïve and primed states have differing biological functions, e.g., developmental potency and chimeric contribution ability [124]. However, most of human iPSC are cultured in a primed state; therefore, much attention focuses on defining the factors (frequently soluble factors) that can revert primed cells to a naïve state [125–129]. A major driver to appreciate the role of culture substrates in pluripotency *continuum* came from the clearly recognizable morphological differences in naïve and primed colonies: naïve cells form dome-shaped 3D colonies, while colonies consisting of primed cells possess a flattened appearance. Despite the lack of information on the effect of growth substrates on the pluripotency status, suppression of ECM-integrin signalling has been linked to the maintenance of naïve human iPSC [130, 131].

Much of the information concerning ESC- and iPSC-integrin interaction stems from the gradual transition of feeder layer-cultured cell lines to more defined matrices such as Matrigel®, Cultrex BME®, Geltrex®, fibronectin, collagen IV, laminins, and vitronectin. A comparison of ESC and iPSC mRNA microarray data revealed that the expression profiles of integrins are similar in both types of pluripotent stem cells. Specifically, *α*5, *α*6, *αν*, *β*1, and *β*5 are all abundantly expressed on iPSC; however, not all iPSC lines displayed identical integrin profiles [132, 133]. Similarly, the integrin *α*3, *α*5, *α*6, *α*9, *αν*, and *β*1 subunits, but not the *α*1, *α*2, *α*4, *α*7, and *α*8 subunits, were identified as markers of undifferentiated porcine-primed ESC, with a subsequent significant increase in their adhesion features on fibronectin, tenascin C, and vitronectin coatings. The blockade of integrin heterodimers *α*5*β*1, *α*9*β*1, and *ανβ*1 lead to a strong inhibition in cell-ECM adhesion [134]. Moreover, *α*ν*β*3, *α*6*β*1, and *α*2*β*1 play a significant role in the initial adhesion of the human ESC to Matrigel [135]. Interestingly, human but not porcine ESC display the active integrin heterodimer *α*6*β*1 [136] suggesting species-dependent differences in the mechanotransduction signalling context. Concerning iPSC features, the parental cell-type origin impacts integrin expression, with enhanced levels of certain integrins observed in iPSC derived from adherent cell types, e.g., foreskin fibroblasts. Interestingly, Rowland and colleagues uncovered important differences between human ESC and iPSC in terms of the essential integrins necessary for initial adhesion and subsequent proliferation on different matrices. Specifically, they showed that *β*1 is necessary for both functions when each cell type was grown on Matrigel® whereas *ανβ*5 and *β*1 are important for iPSC attachment and proliferation when cultured on vitronectin as described in Section 2. Lastly, integrins and integrin-mediated signalling are important in maintaining iPSC self-renewal and pluripotency as indicated by reduced Nanog, Oct-4, and Sox2 levels in *α*6-silenced iPSC lines, localization of the FAK N-terminal domain in nuclei, and AKT signalling activation [136, 137]. Similarly, murine ESC interaction with the RGD peptide plays a role in the expression of core transcription factors, i.e., Oct-4, Sox2, and Nanog. Cyclic RGD synthetic compound supplementation was sufficient to mimic the effect of a mechanical stimulus, in terms of pluripotent gene expression. Specifically, this molecule or mechanical stimulus significantly influenced ESC pluripotency by downregulating core transcription factors. Moreover, RGD peptide, by inhibiting integrin binding and, in turn, integrin expression [6], upregulated early lineage markers (mesoderm and ectoderm) by leukaemia inhibitory factor (LIF) signalling [138]. Interestingly, human ESC, expressing integrin *α*6*β*1, preferentially bind human recombinant laminin-111, laminin-332, and laminin-511, which are good substrates able to maintain undifferentiated pluripotent human ESC cultures [139].

The ultimate destination of iPSC is differentiation along specific cell lineages culminating in the generation of functional terminally differentiated cells. Tailored protocols now exist to generate most cell types from each of the three germ layers, e.g., neurons, pancreatic islet *β*-cells, and, of specific relevance to this review, cardiomyocytes. Subsequently, these iPSC-derived cells can be used to model various diseases and screen novel drugs. Early cardiomyocyte differentiation protocols relied on the appearance of beating clusters within stochastically formed embryoid bodies (EB). The inefficient nature of producing cardiomyocytes from EB leads to the discovery of more efficient methods for cardiogenesis. One option considered here is the employment of small molecules modulating the key stages of embryonic cardiac development, i.e., early mesoderm formation by molecules targeting bone morphogenetic proteins, the wingless/INT (Wnt) proteins, and fibroblast growth factors, followed by activation of the conserved cardiac transcriptional program, i.e., Nkx2.5, Tbx5, Isl1, GATA4, and SRP. This program ultimately leads to the expression of the structural proteins essential for the function of cardiomyocytes, e.g., actin, myosin light/heavy chains, desmin, and the troponins (elegantly reviewed in [140]). Zeng et al. demonstrated that EB growth and cardiac differentiation of EB rely on collagen/integrin *β*1 interaction [141]. Specifically, they observed a synergistic upregulation of collagen and integrin *β*1 which peaked on the third post differentiation induction day [141]. Interestingly, the size and shape of EB as well as the confluence of iPSC are strongly linked to cardiogenic capacity [142–146]. The Wnt pathway is a pivotal pathway strongly linked to iPSC self-renewal and differentiation which is exploited by cardiomyocyte differentiation protocols relying on its temporal activation and inhibition in order to achieve highly efficient cardiomyogenesis [145, 147]. The noncanonical Wnt-planar cell polarity (PCP) pathway is able to induce actin cytoskeleton change promotion through Rac1, RhoA, and small GTPase signalling, which controls cell movement and tissue geometrical features. Good examples are the RhoA signalling cascade activated by DAAM1 and DAAM2 formin homology proteins or the JNK signalling cascade, which is activated by MAPKKK and MAPKK 4/7 [148, 149]. Critically, following prolonged Wnt/*β*-catenin activation, the E-cadherin suppressors, SLUG and SNAIL, act as watershed factors that turn the iPSC fate from self-renewal to committed differentiation [150]. Lastly and more interestingly, for the clinical relevance, is the observation of Zhao and colleagues [151] who showed that the ROCK inhibitor, Y-27632, enhanced the transplantation success (in terms of engraftment) of human iPSC in a murine myocardial infarction model. The same compound revealed positive effects also on human ESC, e.g., increasing migration and supporting differentiation into EB. In the same study, integrin *β*1 blockade abolished the adhesion of ESC which decreased their survival and pluripotent status [152].

### 3.1. Mechanotransduction and Cell Differentiation

The genes under the direct control of the signalling pathways described in Section 3 are multiple. In this section, we will focus on the genes and pathways involved in pluripotent stem cell differentiation into cardiomyocytes.

An interesting 2013 study highlighted how integrin-mediated response to strain can be modulated by cell geometry more than by the cell area. Indeed, given the relevance of cell geometrical cues in mechanotransduction, in several cell types, efficient RhoA activation leads to megakaryocytic leukaemia-1 (MKL-1) protein translocation into the nucleus, in a cell shape-independent manner [153]. MKL-1 is a member of the so-called myocardin-related transcription factor family and physically interacts with the serum response factor (SRF) which activates SRF-dependent downstream gene transcription [154], e.g., actin cytoskeletal/FA-related proteins [155, 156].

In a previous study, it was shown that the skeletal *α*-actin promoter activation, which is downstream of RhoA, was strongly potentiated by *β*1 integrin expression and function. These events were demonstrated to be specifically displayed by cardiomyocytes, but not by NIH 3T3 fibroblasts. This observation further supported RhoA/SRF-dependent cardiomyocyte gene expression by the *β*1 integrin signalling pathway [157]. Concerning the role of SRF in stem cells, the study of murine SRF^−/−^ ESC showed that SRF deficiency causes impairments in cell spreading, adhesion, and migration, due to cytoskeletal structure modifications in terms of actin stress fibres and FA. Moreover, stem cells lacking SRF displayed downregulated FA, FAK, *β*1 integrin, talin, zyxin, and vinculin [158]. Furthermore, depletion of the adhesion molecule integrin *β*3, a key regulator of myogenic differentiation and actin organization, attenuated p130Cas phosphorylation and MKL nuclear localization during myoblast *in vitro* differentiation [50].

The MKL-1/SRF pathway is firmly linked to another important signalling pathway, strongly involved in mechanosensing in cardiovascular cells, namely, yes-associated protein (YAP) and transcriptional coactivator with PDZ-binding motif (TAZ) [159, 160]. Tuning YAP transcriptional activity leads to the modification of cell mechanics, force, and adhesion and determines cell shape, migration, and differentiation [161]. In the last years, this signalling pathway, deeply related to the HIPPO pathway, which is strongly related to developmental biology, is a hot topic in mechanotransduction studies. Indeed, there are numerous papers describing the involvement of YAP/TAZ in osteogenesis [159, 162].

Several studies underlined the indirect role of small GTPase Rho in YAP/TAZ nuclear localization control, exerted by promoting the actin bundles and stress fibre formation in response to cell spreading on the ECM [163–165]. Nardone et al. in 2017 demonstrated that YAP nuclear localization is controlled through Rho/ROCK activation and YAP transcriptionally controls FA formation and cytoskeleton stability which, in turn, determines cell adhesion to the ECM [161].

Experiments on conditional mouse YAP^−/−^ and TAZ^−/−^ in the skin resemble the profibrotic phenotype of skin-specific loss of integrin *β*1, highlighting the strong linkage and interplay of all these molecules *in vivo* [166].

Recently, *β*1 integrin-dependent cell adhesion was seen as a critical element in mesenchymal cell proliferation, both *in vivo* and *in vitro*. In fact, it was demonstrated that *β*1 integrin-dependent activation of the small GTPase Rac1 leads to YAP dephosphorylation and its nuclear shuttling, confirming that *β*1 integrin-dependent Rac1 function plays a key role in YAP regulation, triggered by cell adhesion [167]. Another recent paper identified a pathway involving both activation of integrin *α*3 and a FAK cascade-controlling YAP phosphorylation and thus its nuclear localization in transit-amplifying stem cells. In this work, the authors highlighted that this specific signalling pathway potentiates mTOR signalling, driving cell proliferation, and that the YAP/TAZ signalling mechanism coordinates stem cell expansion and differentiation during organ self-renewal [51].

## 4. Integrin Relevance in iPSC-Derived Cells: In Vitro Biomimetic Approaches

**Figure 3:**
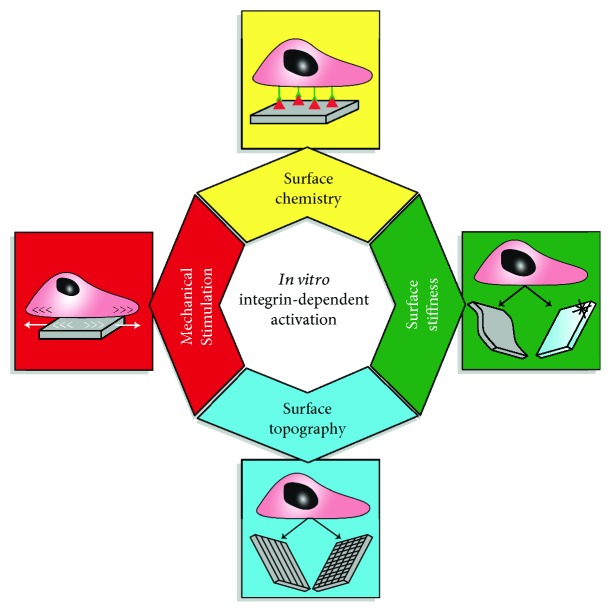
Engineered materials supporting *in vitro* modelling. Integrin-mediated pathways relevant for iPSC cardiac differentiation can be enhanced *in vitro* by the use of ad hoc-designed biomaterials. Toward this aim, chemical, geometrical, mechanical, and physical properties of the substrates are relevant.

As discussed in the first section, mechanosensing, in general and specifically integrin activation, can be used to guide lineage-specific cell fate by activating mechanotransduction pathways. In order to better elucidate the fundamental mechanisms driving pathophysiological mechanisms, several *in vitro* models, based on biomimetic approaches, have been proposed and discussed. This section provides an overview of the *in vitro* models (Figure 3), focusing on the potential of biomimetic approaches to direct iPSC cardiomyocyte differentiation and maturation, possibly supporting their use in the field of cardiovascular regenerative medicine and tissue engineering.

### 4.1. Surface Chemistry

Substrate chemical composition and the motifs decorating a given surface have a strong effect on selective integrin engagement. Here, we will discuss two different approaches to engineering substrates: the first employing ECM obtained by decellularization of biological tissues and the second relying on the functionalization of synthetic biomaterials.

#### 4.1.1. Decellularized ECM

*In vivo*ECM, thanks to its chemical composition and mechanical/topographic properties, establishes the bases to support cell proliferation and differentiation [168, 169]. Indeed, the ECM surface does not only mediate cell attachment by exhibiting anchorage sites for different cell surface receptors and coreceptors but also regulates the diffusion of soluble factors secreted by the neighbouring cells, e.g., ECM composition modifies chemical diffusion coefficient and, as a product of its own remodelling, releases functional fragments constituting additional soluble factors.

For this reason, the use of decellularized tissues, maintaining composition, architecture, mechanical properties, and, interestingly, cell-binding domains, has been widely proposed as a suitable scaffold for *in vitro* cell seeding, expansion, and differentiation [170–174]. In parallel, its specific capacity to guide stem cell differentiation has been also shown [168, 175–177]. In particular, the ability to selectively increase the expression of integrins [171] has been demonstrated, underlining the relevance of ECM protein composition, e.g., the ratio of collagen, fibronectin, laminin, vitronectin [178], and their topology, supporting cell adhesion and subsequently stimulating controlled cell differentiation [171]. The potentiality of scaffolds realized by tissue decellularization is maximized by the use of dynamic culture methods, i.e., perfusion bioreactors supporting homogeneous repopulation of the whole scaffold volume [179–181] and recreating a controlled and reproducible 3D environment.

Nevertheless, although experiments are performed under highly controlled culture conditions, the coexistence of multiple parameters limits the understanding of the impact of specific factors. Therefore, decellularized ECM scaffolds are good multifactorial model systems, comprehensive of the complexities of the *in vivo* scenarios, and are suitable for translational studies. On the other hand, biomaterials have been designed and functionalized ad hoc, by means of either coating with ECM components or generating specific cell-binding domains, in order to interpret different mechanisms.

The intermediate link in this chain is constituted by 3D bioprinting technologies. The technological advances in the field have allowed the use of liquefied decellularized tissues for high-resolution precise simulation of native tissue structures, with encapsulated cells [182, 183], thus providing the chance to observe the biological effects induced by fine tuning of local chemical/architectural matrix modifications.

#### 4.1.2. Engineered Biomaterials

Polymers, both synthetic and natural, have been widely used for the manufacture of substrates and scaffolds intended for in vitro cell culture and cardiovascular tissue engineering [184]. The most used synthetic polymers in the field are poly(ethylene-glycol) (PEG), poly(lactic-acid) (PLA), poly(glycolic-acid) (PGA), poly(*ε*-caprolactone) (PCL), and their copolymers such as poly(lactide-co-glycolide) (PLGA) and polyurethanes (PU) [185–190]. In contrast, natural materials include ECM constituents, i.e., collagen, fibrin, and silk [178, 191, 192]. The advantages of using engineered biomaterials rely on their amenability to fine-tune parameters, such as biocompatibility, local rigidity, micro/nanoarchitecture, and functionalization with ECM proteins, integrin-binding peptides, or growth factors [186, 193].

By coating synthetic materials, e.g., PU, with fibronectin (REDV, PHSRN, RGD, and GRGDSP), laminin (IKLLI, IKVAV, LRE, PDSGR, RGD, and YIGSR), and collagen (DGEA) sequences [194–196] together with supplementing cell culture media with selective integrin inhibitors [197], the effect of integrin expression on cell attachment and proliferation has been highlighted. In addition, polymer functionalization with ECM peptides has been proposed to actively promote cell differentiation. An example, performed with an elegant and innovative approach, has been proposed by Ovadia and colleagues [198]. Matrigel®, a commercial solubilized basement membrane preparation extracted from the Engelbreth-Holm-Swarm mouse sarcoma cells, consisting of laminin, collagen IV, proteoglycans, and a number of growth factors, is widely recommended as an iPSC culture support. However, the specific reasons behind its success have not been elucidated. In their work, Ovadia et al. proposed iPSC single-cell encapsulation within 3D photopolymerized with UV light (365 nm for 2 minutes) PEG-peptide-based synthetic gels conveniently functionalized by a range of motifs inspired by Matrigel and known to bind a variety of integrins (including *α*6, *αν*, and *β*1), which generally promotes cell adhesion. Knowing that ROCK inhibition increases the expression of *αν*, *α*6, and *β*1 integrins during iPSC culture on Matrigel®, in the proposed 3D culture system, the authors showed that iPSC viability, growth, and differentiation were enhanced in response to the environment, in particular to *β*1 integrin activation.

Another parameter that needs to be taken into account when modelling cell adhesion *in vitro* is the coupling strength between integrin ligand and substrate. Human mesenchymal stem cells exhibit enhanced osteogenesis in response to high-strength binding which results from the activation of a YAP-mediated pathway [199]. This result suggests that traditional covalent biological coatings could generate a bias in data interpretation, which implies the need for novel coating methods, such as noncovalent coatings [200]. Based on these findings, it is clear that the study of integrin recruitment can not omit the consideration of forces acting on the whole chain, from the substrate, e.g., substrate rigidity to the engaged cytoskeleton filaments. Combinatorial approaches have been implemented for the production of copolymers able to tune cell-substrate interaction. An example of this approach is provided by Cheng et al. [201], who demonstrated that novel supramolecular PCL-containing self-complementary sextuple hydrogen-bonded uracil-diamidopyridine moieties can positively support cell attachment and proliferation. Moreover, as demonstrated by Chun et al. in 2015 [202], the copolymer generated by polymerization of monomeric *ε*-caprolactone with methoxy-PEG, in the ratio of 4% PEG-96% PCL, was able to enhance iPSC-d-CM contractility, upregulating the expression of mature cardiomyocyte markers such as myosin light chain-2*ν* and cardiac troponin I. The authors demonstrated that these effects are linked to the engagement of a subset of integrins which activate a mechanosensory transduction pathway regulated by the polymerization of intermediate filaments.

### 4.2. Surface Topography

*In vivo* cardiac tissue functionality is aided by the anisotropic tissue structure which results from ECM protein organization, cell orientation, and cell-ECM/cell-cell junctions. *In vitro*, the relevance of ECM architecture in iPSC maturation has been demonstrated by means of micro- and nanostructured substrates. Results demonstrated how geometrical cues could support iPSC pluripotency and differentiation [203, 204] by the formation of cell-cell junctions, not only leading to the generation of more functional grafts, increased beating rate, and enhancing tissue-specific protein arrangement, e.g., sarcomeric-actinin, connexin 43, and troponins, but also allowing better stratification of the pathology, e.g., muscular dystrophy [203, 205–207].

At the aim of understanding the involvement of integrins in geometrical feature-driven cell differentiation, substrates controlling either cell shape and size or cell alignment or spacing of adhesion ligands have been designed.

#### 4.2.1. Cell Alignment

*In vivo*, physiological cardiac functionality is supported by coordinated muscular contraction, which is allowed by highly organized cell alignment, guaranteeing controlled anisotropic conduction of the electrical stimuli. *In vitro* iPSC-d-CM assemble in heterogeneous randomly organized clusters, missing accurate reproduction of the *in vivo* scenario. This limits their level of maturation and excludes from the *in vitro* model the effects of possibly relevant mechanotransduction-guided mechanisms. The introduction of nanotopographical features into culture substrates, i.e., grooves in the 700–1000 nm range [11], has been demonstrated to improve cardiomyocyte development by acting on one hand through a reorganization of the integrin activation of the single iPSC (i.e., enhancing integrin expression and formation of FA and increasing F-actin polymerization) and, on the other, geometrically organizing the colony polarization. Culturing on grooved substrates finally impacts on iPSC-d-CM intrinsic molecular machinery, i.e., through the activation of the YAP-dependent pathway [208, 209], resulting in more physiological behaviours, showing a reduction of arrhythmias and inducing more mature Ca^2+^ spark patterns [210, 211]. The maturity level not only would benefit from cell alignment but could permit a more significant stratification of the pathology, as demonstrated by the limited capacity of iPSC-d-CM from patients affected by Duchenne muscular dystrophy versus healthy donors in their ability to reorient when cultured on grooved substrates [212].

More recently, the introduction of polymeric nanowires offers the chance to couple cell alignment with electrical conductivity and stimulation, reproducing preferential routes for geometrical organization and electrical signal timing. The application of this technology resulted in a significantly more advanced cellular structure, i.e., showed by cell-cell junction formation, and contractile function efficiency [213–215], enhancing *in vitro* iPSC-d-CM maturation and functionality and leading towards the design of a better model system for the evaluation of the *in vivo* pathophysiological mechanisms.

#### 4.2.2. Cell Shape and Size

In between, the geometrical features able to guide *in vitro* stem cell differentiation, shape, and size have been widely studied [216]. Indeed, several screening platforms, also commercial platforms, e.g., BioSurface Structure Array, Nano-TopoChip [217], have been used to demonstrate that by regulating the width/length ratio, in the presence or absence of soluble factors, cell fate can be moved from osteogenic to adipogenic commitment.

Regarding iPSC, they are often cultured as aggregates and not as single cells; therefore, the mechanotransduction-driven effects of whole-colony size and shape should be taken into account. Indeed, by controlling colony size, density, shape, and spacing, Myers et al. [218] improved homogeneity in the expression of pluripotency markers (SSEA4 and Nanog). Moreover, the proposed micropatterning technique, through the standardization of cell density, increased the percentage of spontaneous beating cells. In particular, the generation of circular patterns leads to the formation of connecting rings of cardiomyocytes, supporting *in vitro* physiological electrical behaviour, i.e., supporting the propagation of contractile waves throughout the ring. Another example of how cell shape, coupled with the supracellular structure, can be used to promote *in vitro* cardiomyocyte maturation is provided by the work of Xu et al. [211]. Here, by imposing single-cell elongation by culturing on silicon-patterned substrates, FA can be regulated to support alignment and cell-cell contacts leading to increased cardiac differentiation efficiency. Moreover, in their recent work, Grespan and colleagues [203] cultured iPSC onto microstructured (square micropillars) silicon substrates and observed that, while not affecting pluripotency, nuclear deformability is sensibly regulated during germ layer specification, happening during iPSC differentiation.

These observations, taken together, finally call for the design of more complex *in vitro* substrates, taking into account mechanosensing mechanisms for better iPSC differentiation.

#### 4.2.3. Integrin Clustering Methods

The methods described in the previous paragraphs are based either on functionalization of substrates by random decoration with integrin-binding domains or by induction of adhesion sites by geometrical constraint and do not encompass the relevance of integrin geometrical distribution. It has been made possible to involve this aspect thanks to the development of novel nanotechnologies, which can be divided in three different approaches [219]: (i) blending of polymers with different degrees of ligand incorporation [220, 221], (ii) nanoprinting lithography of nanoparticle arrays [222–224], and (iii) transfection of proteins by chimera constructs [225]. Results demonstrated that not only identity, abundance, and density of adhesion sites but also their spatial confinement, including global and local density, regulate cell adhesion [226], migration, proliferation, and differentiation acting on both cell-substrate and cell-cell contact [224]. Furthermore, the capacity of nanoscale spatially organized cell-adhesive ligands to direct stem cell fate was also demonstrated [210, 227].

### 4.3. Surface Elasticity

Substrate stiffness has been shown to be a very strong mechanotransduction stimulus, regulating physiopathological cell behaviour and cell reprogramming and subsequently guiding the development of mature cell phenotypes [164, 228–233]. In particular, regarding the *in vitro* application of iPSC technology in the cardiac field, matrix rigidity can guide iPSC-d-CM differentiation: the use of a substrate with compliance similar to that of native cardiac tissue [234–236] supports cardiac commitment and enhances metabolic maturity, sarcomeric protein subtype, cardiac troponin T expression, and force generation [230, 235, 237, 238]. The molecular events transferring the force from the substrate to the nuclei, through cytoskeleton engagement, have been described by Zhou et al. [239], and other reviews discussed this topic at length [240, 241]. Here, we will underline some specific aspects about the involvement of integrins in this phenomenon. Indeed, the selective switching from the activation of *β*3 to *β*1 integrins in response to reduced substrate stiffness has been demonstrated [197, 242, 243]. From a technological point of view, it is interesting to underline the sensitivity of the whole traction chain to integrin-substrate binding force. Indeed, a modification in the substrate-anchoring strength of integrin-binding ligands, i.e., choosing covalent binding to obtain stable substrate coating, could lead to a misinterpretation of *in vitro* cell behaviour [15, 199, 244, 245], thus highlighting the importance of considering mechanical stimuli, i.e., surface elasticity, with the feeling of cells, recognizing the role of all the nanoscale players.

### 4.4. Mechanical Stimulation

Mechanical stimulation has been demonstrated to regulate FA assembly, modulating the downstream pathways affecting cardiomyogenesis [246–249]. Based on this assumption, several methods have been described for the application of controlled mechanical stimulation (i.e., temporal, spatial, and amplitude), some of them aiming to verify the positive impact of integrin-mediated adhesion pathways on iPSC reprogramming [113] and differentiation [250, 251]. Although far from being exhaustively described, the pathways seem to be regulated by the change in FA density and local conformation [252], followed by impacting cytoskeleton rearrangement [56], finally regulating cardiomyocyte maturity, e.g., cell-cell contact, sarcomeric structure, and electrical activity. As an example, the interconnection between the mechanical stimulation, in particular shear stress, and the modulation of cellular electrical activity was demonstrated by Roy and Mathew [253], who underlined how the gene encoding the *α*-subunit of human ether-a-go-go-related gene (hERG) potassium ion channel could be modulated by integrins via a mechanoelectric feedback pathway. Not only that mechanical stimulation enhances cell electrical behaviour but that a positive effect on maturation of cardiomyocytes *in vitro* has been demonstrated by coupling pacing with mechanical stimulation [254]. Finally, the *in vitro* implementation of mechanical stimulation has been shown of benefit in the model for the pathology stratification. Chun and colleagues proposed [249] that the application of cyclic or static strain modulated the gene expression of a cell-cell connection-related protein (connexin-43) in iPSC-d-CM which was more pronounced in iPSC-d-CM from patients affected by primary dilated cardiomyopathy.

## 5. Conclusions

Each integrin type is coupled to a different combination of signalling cascades which drive specific cellular processes, e.g., stem cell differentiation [255, 256]. Integrins are involved not only in the recognition of substrate composition but also in sensing ECM rigidity and adapting cell morphology, motility, and fate to the mechanical properties of the matrix, through the activation of mechanotransduction pathways.

However, the high specificity in the link between integrin-mediated cell response to tissue-specific microenvironment is far from being completely decrypted, both in general [15] and more specifically in pluripotent stem cells [98]. A detailed understanding of the cellular machinery linking mechanosensing to mechanotransduction would be beneficial for effective *in vitro* modelling and the future clinical translation of tissue-specific differentiated iPSC.

As discussed in this review, the implementation of *in vitro* novel biomaterials, taking into account integrin-mediated mechanotransduction signalling, coupled with controlled systems, i.e., microfluidic bioreactors, could be relevant to improving the study of iPSC-d-CM differentiation and supporting maturation [12, 257], inspired by an “organ/lab-on-chip” approach [227]. The design of such models would benefit (i) *in vitro* modelling of the molecular basis of the pathologies, (ii) *in vitro* evaluation of possible mechanisms and specific molecular targets for personalized pharmacological approaches, and (iii) development of a mature cell source available for future transplantation perspectives. Indeed, the high risk of teratoma formation intrinsic to transplantation of iPSC-derived cells is well acknowledged. Moreover, the maturity of the implanted cells, especially thinking about cardiac applications of iPSC-d-CM, should guarantee their survival and functionality shortly after the procedure.

In conclusion, these aspects would raise the level of iPSC-d-CM quality and provide an effective model system for the study of different cardiac pathologies. Moreover, in an optimal scenario, the use of bioactive scaffolds in controlled culture systems could permit the utilization of read-out parameters that provide a culture quality feedback signal.

## Figures and Tables

**Table 1:** Integrin heterodimers, extracellular ligands and downstream signalling pathways.

	Integrin heterodimers	Ligands	Pathway	Ref.
Collagen receptor	*α*1*β*1	Collagen (IV, I, and IX)	(i) RhoA/ROCK	[51]
	*α*2*β*1	Collagen (I, IV, and IX)	(i) RhoA/ROCK (ii) YAP/TAZ	[52]
	*α*10*β*1	Collagen (IV, VI, II, and IX)	(i) RhoA/ROCK	[51]
	*α*11*β*1	Collagen (I, IV, and IX)	(i) RhoA/ROCK	

Laminin receptor	*α*3*β*1	Laminin (LN-511, LN-332, and LN-211)	(i) RhoA/ROCK	[51]
	*α*7*β*1	Laminin (LN-511, LN-211, LN-411, and LN-111)	(i) RhoA/ROCK	
	*α*6*β*1	Laminin (LN-511, LN-332, LN-111, and LN-411)	(i) RhoA/ROCK	
	*α*6*β*4	Laminin (LN-332, LN-511)	/	

RGD receptor	*ανβ*1	Fibronectin, vitronectin (RGD)	(i) RhoA/mDia	
	*α*ν*β*3	Vitronectin, fibronectin, and fibrinogen (RGD)	(i) RhoA/mDia (ii) MKL-1/SRF (Responsible for the increase in the number of FA and cell spreading areas)	[17, 18, 50, 53]
	*α*ν*β*5	Vitronectin (RGD)	(i) RhoA/mDia (Responsible for the increase in the number of FA and cell spreading areas)	[17, 53]
	*α*ν*β*6	Fibronectin, TGF-*β*-LAP (RGD)	/	
	*α*ν*β*8	Vitronectin, TGF-*β*-LAP (RGD)	/	
	*α*5*β*1	Fibronectin (RGD)	In the leading edge of moving cells in the 2D surface	[16]
	*α*8*β*1	Fibronectin, vitronectin, and nephronectin (RGD)	/	
	*α*IIb*β*3	Fibrinogen, fibronectin (RGD)	/	

Leucocyte-specific receptor	*α*9*β*1	Tenascin-C, VEGF-C, and VEGF-D	/	
	*α*4*β*1	Fibronectin, VCAM-1 (LDV)	/	
	*α*4*β*7	MadCAM-1 (LDV), fibronectin, and VCAM-1	/	
	*α*D*β*2	ICAM-3, VCAM-1	/	
	*α*E*β*2	E-cadherin	/	
	*α*L*β*2	ICAM-1, ICAM-2, ICAM-3, and ICAM-5	/	
	*α*M*β*2	iC3b, fibrinogen	/	
	*α*X*β*2	iC3b, fibrinogen	/	
